# Effects of insomnia on symptomatic dry eye during COVID-19 in China: An online survey

**DOI:** 10.1097/MD.0000000000035877

**Published:** 2023-11-17

**Authors:** Guanghao Qin, Xiaoying Luan, Jiayan Chen, Liangzhe Li, Wei He, Emmanuel Eric Pazo, Xingru He, Sile Yu

**Affiliations:** a He Eye Specialist Hospital, Shenyang, China; b He University, Shenyang, China.

**Keywords:** COVID-19, dry eye, insomnia, online survey

## Abstract

Sleep is an essential determinant of health and quality of life. This study aimed to evaluate sleep disorders and symptomatic dry eye (DE) occurrence. This study was a cross-sectional survey of 1393 participants in China. The insomnia severity index (ISI) Questionnaire was used to evaluate sleep quality, and the ocular surface disease index (OSDI) questionnaire was used to assess DE symptoms. Subjects were divided into 2 groups based on subjects with and without symptomatic DE. The patients who had DE (10.48 ± 7.27) had substantially lower ISI scores compared to those without DE (3.57 ± 5.10) (*P* = .003). Furthermore, each ISI item and total ISI score was significantly correlated with OSDI dry eye severity and total OSDI dry eye score. Higher prevalence of insomnia was found in participants with symptomatic DE, and insomnia correlated significantly with DE symptoms. The present results suggest that clinicians and healthcare workers need to remember that DE and insomnia are highly co-existing health issues.

## 1. Introduction

Dry eye disease (DED) is a multifactorial chronic ocular surface disease defined as a tear film disorder due to tear deficiency or excessive tears evaporation.^[1]^ Dry eye (DE)-related symptoms (DES), including irritation, eye burning, tired eyes, ocular dryness, tearing, foreign body sensation, and eye discomfort, make one of the main groups of “computer vision syndrome” symptomatology.^[2]^ The increased use of digital screens following the COVID-19 pandemic has increased the prevalence of DED, especially among adolescents; the prevalence of DED has been reported to be as high as 70.5%.^[3]^ DE related to digital screen has been postulated to result in tear film instability due to reduced blink rate and incomplete eyelid closure.^[4]^ Furthermore, since the COVID-19 pandemic, traditional learning and working methods have changed in various countries due to online learning courses and remote working. Furthermore, work and leisure activities have also been altered following the epidemic.^[5,6]^ Studies have reported increased stress and insecurities regarding social and economic status.^[7]^ Additionally, the subsequent increased use of digital social networks has reduced outdoor activities, daylight exposure, and exercise, thereby exacerbating sleep rhythm and patterns.^[8,9]^ While the alterations mentioned above to lifestyle are likely to affect sleep characteristics in terms of quantity and quality, there is a paucity of research regarding the use of sleep-specific questionnaires and DED during the pandemic.^[10–12]^

Currently, in China, the impact of digital screens on myopia in children and adolescents has garnered attention^[13–15]^ and the increased exposure to digital screens has aggravated other ocular surface and psychological issues, such as DE and insomnia.^[3,16–18]^ Recent studies have reported that DED symptoms often accompany systemic comorbidities such as migraine, chronic pelvic pain, musculoskeletal pain, chronic pain conditions, irritable bowel syndrome, insomnia symptoms, and low quality of life.^,[8,9,11,12]^ Both DED and sleep disorders have been implicated in reducing the quality of life, and recent studies have pointed to a possible relationship between sleep disturbance associated with circadian rhythm disruption and even hypertension and metabolic syndrome.^[17–20]^ It has been reported that tear secretion and tear stability have a circadian rhythm^[21,22]^ and tear secretion system might be associated with the renin–angiotensin system,^[23]^ as metabolic syndrome patients have lower tear secretion.^[20]^

We conducted the present study to explore the relationship between poor sleep quality and DED. Our research aims to explore the relationship between the various components of DE symptoms and sleep disorders during the COVID-19 pandemic in China.

## 2. Methods

### 2.1. Study design and participants

This was a cross-sectional study involving Chinese participants from July to August 2022. The study protocol was approved by the Institutional Review Board of He Eye Specialist Hospital, Shenyang, China (approval no: IRB (2022) K018.01) and conducted according to the ethical standards in the Declaration of Helsinki.

Web-based questionnaires were administered via the WeChat internet platform (WeChat instant messaging app, Shenzhen, China). The general question domain consisted of the following questions: age (year), gender (Male/Female), profession (Student, Teacher, Worker, Farmer, Medical work, Company employee, Civil servant, Military personnel, Freelancers, Other), and daily screen time (<1 hour, 1–2 hours, 2–4 hours, 4–6 hours, ≥6 hours). The insomnia severity index (ISI) Questionnaire was used to evaluate sleep quality, and the ocular surface disease index (OSDI) questionnaire was used to examine the prevalence of DE among participants.

### 2.2. OSDI questionnaire

A web-based Chinese version of OSDI (Allergan Inc, Irvine, CA) was used to assess DE and provided a quantifiable assessment of DE symptom frequency and the impact of these symptoms on visual function validated by Zhang et al in 2021.^[19]^ It contains 12 items, and the score can range from 0 (no symptoms) to 100 (severe symptoms) points; 0 to 12 represents normal, 13 to 22 represents mild DED, 23 to 32 represents moderate DED, and >33 represents severe DED).

### 2.3. ISI questionnaire

Insomnia Severity Index (ISI), is a concise self-assessment questionnaire that has been previously demonstrated to possess both reliability and validity. It serves as an instrument for quantifying an individual perceived severity of insomnia.^[20]^ In this study, a Chinese version of the ISI questionnaire validated by Yu^[21]^ and Chung et al^[22]^ was used to evaluate sleep quality. It is a 7-item scale assessing the perceived severity of insomnia symptoms (initial, middle, terminal), the degree of satisfaction with sleep, interference with daytime functioning, noticeability of impairment, and concern caused by sleep problems.^[23]^ Each item is rated on a 0 ± 4 scale; the total score ranges from 0 to 28. Insomnia severity is classified by ISI into 4 categories: 0 to 7 points represent absence of insomnia, 8 to 14 points represent subthreshold (mild) insomnia, 15 to 21 points represent moderate insomnia, and 22 points or more represent severe insomnia (22 points or more). In the present investigation, insomnia was defined as an ISI score of more than or equal to 8. A greater score indicates a higher degree of insomnia severity.^[20]^

### 2.4. Statistical analyses

The characteristics of the study population were evaluated using descriptive statistics. Logistic regression models were used to assess the relationship between poor sleep quality (ISI score ≥ 8 as the dependent variable) and dry eye (“dry eye” OSDI score > 12 as the independent variable). In addition, to investigate which components of ISI questionnaire items of sleep quality were correlated separately with DE. A *P* value lower than .05 was considered statistically significant in all analyses above.

## 3. Results

Study characteristics are listed in Table 1 and describe the characteristics of the study population (n = 1393) comprising 61% females. The mean age of the participants was 34.36 ± 12.79 years, and mean OSDI score was 28.66 ± 23.83, and the ISI score was 8.38 ± 7.40.

**Table 1:** Population characteristics.

	Mean	Std. deviation
Age (yr)	34.36	12.79
Sex (F/M)	849 (61%)/ 544 (39%)	
Height (m)	1.92	6.52
Weight (kg)	60.92	12.17
BMI (kg/m^2^)	21.70	3.67
Total OSDI Score (0–100)	28.66	23.83
Total ISI Score (0–28)	8.38	7.40

Data presented as mean, standard deviation, and n (%).

BMI = body mass index, F = female, ISI = insomnia severity index, kg = kilogram, M = male, m = meters, OSDI = ocular surface disease index.

Table 2 compares total mean OSDI scores and total mean ISI scores between the sexes. Males were found to have higher total mean OSDI score and total mean ISI score in comparison to females (29.00 ± 24.80 vs 28.44 ± 23.10 and 8.90 ± 7.44 vs 8.06 ± 7.37 respectively). However, both differences were not significantly different (*P* > .05).

**Table 2:** Questionnaire total mean score comparison.

	OSDI total score (0–100)	ISI total score (0–28)
Female	28.44 ± 23.10	8.06 ± 7.37
Male	29.00 ± 24.80	8.90 ± 7.44
*P* value	.665	.043
F value	0.188	4.095

Data presented as mean, and standard deviation.

ISI: insomnia severity index, OSDI: ocular surface disease index.

**Figure 1.**
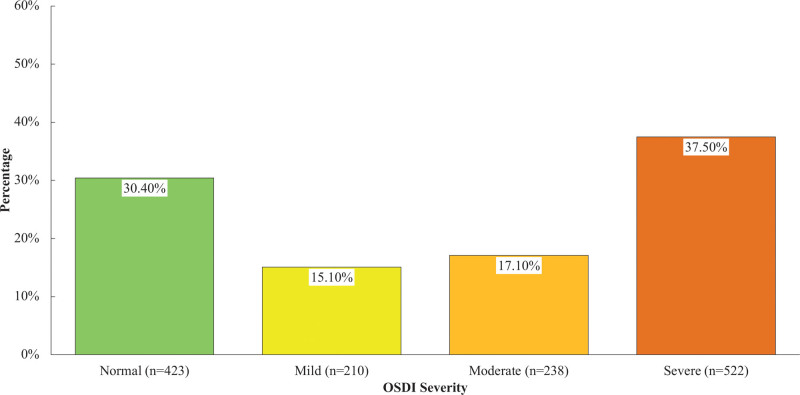
Distribution of OSDI severity.

Figure 1 illustrates the percentage distribution of DE severity among the participants. Severe DE participants were found to be the majority at 37.50%, followed by normal (30.4%), moderate DE (17.10%), and mild DE (15.10%).

**Figure 2.**
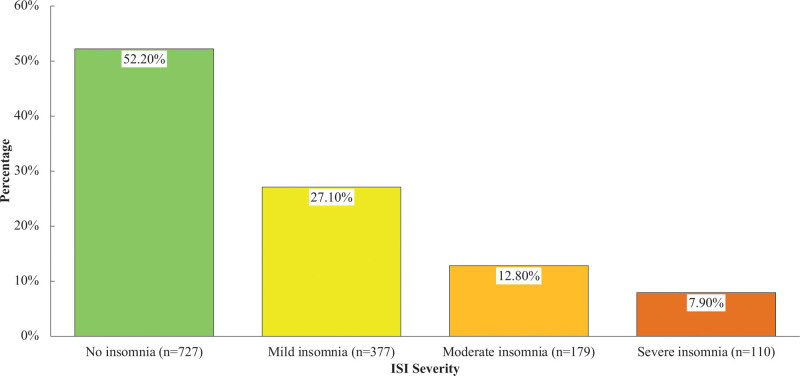
Distribution of ISI severity.

The severity of insomnia among the participants is displayed in Figure 2. Most participants were found not to have insomnia (52.20%), and a minority reported severe insomnia (7.90%).

**Figure 3.**
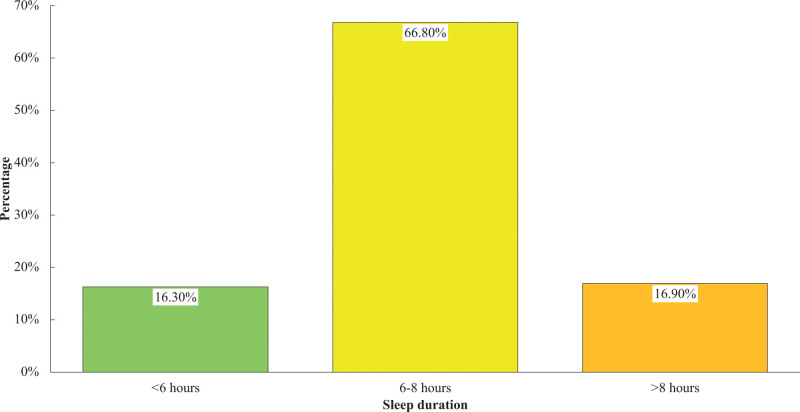
Distribution of sleep duration.

Figure 3 shows a majority of participants slept 6 to 8 hours (66.80%), followed by >8 hours (16.90%), and finally, <6 hours (16.30%) was found to be a minority.

**Figure 4.**
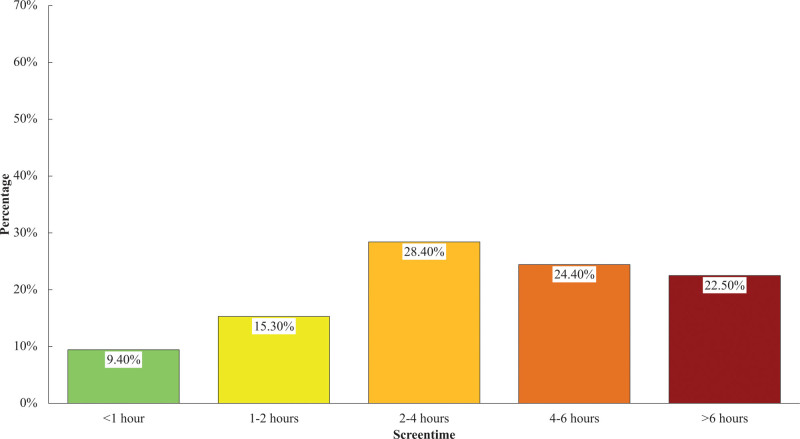
Distribution of screentime.

The distribution of screen time reported by participants is shown in Figure 4. Two to 4 hours (28.40%) was found to be the majority, followed by 4 to 6 hours (24.40%), >6 hours (22.50%), 1 to 2 hours (15.30%), and <1 hours (9.40%) respectively.

Table 3 shows the correlation between the 7 ISI questionnaire items, OSDI dry eye severity, and OSDI dry eye total score. All 7 ISI questionnaire items were found to significantly (*P* < .05) correlate with the OSDI severity score. Additionally, these items were found to correlate significantly (*P* < .05) with the total OSDI score.

**Table 3:** Correlation between ISI questionnaire items with OSDI severity and score.

		ISI 1	ISI 2	ISI 3	ISI 4	ISI 5	ISI 6	ISI 7
OSDI severity scale	Correlation	0.472**	0.491**	0.465**	0.488**	0.491**	0.519**	0.345**
	*P* value	<.001	<.001	<.001	<.001	<.001	<.001	<.001
OSDI total score	Correlation	0.583**	0.616**	0.558**	0.600**	0.608**	0.616**	0.412**
	*P* value	<.001	<.001	<.001	<.001	<.001	<.001	<.001

ISI = Insomnia Severity Index, OSDI = ocular surface disease index.

**P < 0.001.

Table 4 shows the ISI item difference between normal and DE. A significant difference was found between the 2 groups (normal and DE) with all items of the ISI questionnaire.

**Table 4:** ISI item difference between normal and dry eye.

		Mean	Std. deviation	95% confidence interval	
ISI 1	Normal	0.50	0.82	0.33	0.66
	Dry eye	1.25	1.16	1.11	1.40
*P* value		<.001			
F value		35.766			
ISI 2	Normal	0.39	0.73	0.24	0.53
	Dry eye	1.13	1.19	0.98	1.28
*P* value		<.001			
F value		34.266			
ISI 3	Normal	0.42	0.79	0.26	0.57
	Dry eye	1.15	1.15	1.00	1.29
*P* value		<.001			
F value		33.661			
ISI 4	Normal	0.49	0.94	0.30	0.67
	Dry eye	1.33	1.19	1.18	1.48
*P* value		<.001			
F value		40.669			
ISI 5	Normal	0.39	0.82	0.22	0.55
	Dry eye	1.33	1.25	1.17	1.49
*P* value		<.001			
F value		48.822			
ISI 6	Normal	0.42	0.83	0.25	0.58
	Dry eye	1.56	1.21	1.40	1.71
*P* value		<.001			
F value		74.541			
ISI 7	Normal	1.04	0.97	0.85	1.23
	Dry eye	1.73	1.10	1.59	1.87
*P* value		<.001			
F value		30.116			
ISI severity	Normal	1.22	0.54	1.11	1.32
	Dry eye	1.88	0.91	1.76	2.00
*P* value		<.001			
F value		46.345			
ISI total score	Normal	3.62	5.06	2.63	4.62
	Dry eye	9.48	6.92	8.60	10.36
*P* value		<.001			
F value		59.07			

Data presented as mean, standard deviation.

ISI = insomnia severity index, OSDI = ocular surface disease index.

### 3.1. Discussion

The present study ascertained that insomnia symptoms and severity correlated significantly associated with DE symptoms. The most notable finding was that individuals diagnosed with DE syndrome exhibited the most unfavorable ratings on the Insomnia Severity Index (ISI). A previous study pointed out that insomnia and short sleep duration are both significantly correlated with symptoms of DE,^[24]^ which was consistent with our research. Similarly, a study on veterans reported that DE symptom severity is positively associated with insomnia severity.^[25]^

Several reports have indicated a strong correlation between DE and depression or a decline in quality of life. This association has been found to significantly impact productivity among working people.^[26–28]^ One possible cause could be the interconnectedness between persistent stress and the experience of dryness or irritation on the ocular surface. Notably, the symptoms and severity of insomnia in the DE group were significantly higher in the DE group compared to the non-DE group. We hypothesize that individuals with DE are frequently concerned about their eyes because the symptoms are lifelong, of unknown origin, and can worsen at any time due to environmental, seasonal, or local/systemic problems.^[29]^ As a result, DE can produce continual discomfort or anxiety, leading to mood disorders, particularly in elderly people who are more sensitive to unpleasant emotions and feelings of helplessness. Blindness due to eye disease may result in affective disorders, whereas sleep issues are commonly associated with mood disorders. This is consistent with the observation that sleep, and mental issues predominated in DE patients. However, additional research is needed because the cross-sectional study design and inclusion procedure used in this survey did not allow the following questions to be answered: Do sleeping disorders cause DE, or does sleeping disorders cause DE? Is the DE in these people caused by sleep medications?

This study had some limitations. Due to the exploratory survey nature of this research, the clinical diagnosis of DE was not explored in this study. Therefore, the results need to be further evaluated to clarify the relationship between insomnia and clinical DE diagnosis. The current study findings are correlational, and causation hypotheses would necessitate evaluations at different time points. Besides, our results based on the questionnaire need to be further verified in future studies. To mitigate potential confounding factors arising from systemic comorbidities, it is important to exclude patients with cataract, glaucoma, vision impairment, and other ocular comorbidities that may impact sleep and mood when comparing individuals with DED to those without DED. This exclusion would help ensure that the study findings are not impacted by these specific ocular conditions. Pathologies such as cataract^[30]^ and glaucoma^[31]^ can potentially exacerbate sleep and mood and have revealed considerable associations between eye diseases and psychiatric status. Glaucoma is a prevalent eye disease that requires lifelong topical medication, which has known ocular surface toxicity^[32]^ and is often complicated by DED. While various systemic diseases, conditions, drugs, and surgeries might exacerbate signs and symptoms of DE, the scope of this study was not to explore the causative factors that lead to DE.

In conclusion, a significantly higher prevalence of insomnia and correlation was found in patients with symptomatic DE. The holistic management of visitors to an eye clinic should encompass advice on sleep problems, as using such measures would enhance the overall quality of healthcare provided to these individuals.

## Acknowledgments

We thank the participants in this study. This manuscript has not been published and is not under consideration for publication elsewhere.

## Author contributions

**Conceptualization:** Liangzhe Li, Xingru He, Sile Yu.

**Data curation:** Xiaoying Luan, Liangzhe Li.

**Formal analysis:** Xiaoying Luan.

**Funding acquisition:** Xingru He, Sile Yu.

**Investigation:** Wei He, Xingru He, Sile Yu.

**Methodology:** Guanghao Qin, Sile Yu.

**Project administration:** Wei He, Xingru He, Sile Yu.

**Resources:** Jiayan Chen, Wei He, Emmanuel Eric Pazo, Sile Yu.

**Software:** Jiayan Chen.

**Supervision:** Emmanuel Eric Pazo, Xingru He, Sile Yu.

**Validation:** Liangzhe Li, Emmanuel Eric Pazo, Xingru He.

**Writing – original draft:** Guanghao Qin.

**Writing – review & editing:** Emmanuel Eric Pazo, Xingru He, Sile Yu.
